# Analysis of the Composition of Different Instars of *Tenebrio molitor* Larvae using Near-Infrared Reflectance Spectroscopy for Prediction of Amino and Fatty Acid Content

**DOI:** 10.3390/insects14040310

**Published:** 2023-03-23

**Authors:** Nina Kröncke, Stefan Wittke, Nico Steinmann, Rainer Benning

**Affiliations:** 1 Institute of Food Technology and Bioprocess Engineering, University of Applied Sciences Bremerhaven, An der Karlstadt 8, 27568 Bremerhaven, Germany; 2 Laboratory for (Marine) Biotechnology, University of Applied Sciences Bremerhaven, An der Karlstadt 8, 27568 Bremerhaven, Germany

**Keywords:** nutritional composition, near-infrared reflectance spectroscopy, edible insects, *Tenebrio molitor*, instars, harvest time

## Abstract

**Simple Summary:**

Insects are receiving increasing attention as an important protein source that is being proposed as an alternative to fish and soy meal in livestock feed. The aim of this study was to analyze the composition of mealworm larvae at different stages of larval instar using a spectroscopic method for the prediction of the amino and fatty acid content and to search for the optimal harvesting time. Water, protein and fat content, in particular, as well as the amino acid and fatty acid composition were the focus of these investigations. The results of this research revealed that the composition of the different larval instars varies greatly. Moisture and protein content decreased in line with the developmental stages, and their minimum content was recorded in the last instar. By contrast, fat content was lowest in the first instars and increased with larval development. There is a reduction in growth in later instars, meaning that an earlier instar stage is favorable for harvesting. It was possible to predict the amino acid and fatty acid content of mealworm larvae with high accuracy. This can help insect producers to detect the nutrient composition of the larvae easily and quickly so that they can modify larval composition with regard to the amino and fatty acid content to improve the rearing conditions in terms of feeding, while ensuring a stable product quality.

**Abstract:**

Insects are a sustainable protein source for food and feed. The yellow mealworm (*Tenebrio molitor* L.) is a promising candidate for industrial insect rearing and was the focus of this study. This research revealed the diversity of *Tenebrio molitor* larvae in the varying larval instars in terms of the nutritional content. We hypothesized that water and protein are highest in the earlier instar, while fat content is very low but increases with larval development. Consequently, an earlier instar would be a good choice for harvest, since proteins and amino acids content decrease with larval development. Near-infrared reflectance spectroscopy (NIRS) was represented in this research as a tool for predicting the amino and fatty acid composition of mealworm larvae. Samples were scanned with a near-infrared spectrometer using wavelengths from 1100 to 2100 nm. The calibration for the prediction was developed with modified partial least squares (PLS) as the regression method. The coefficient for determining calibration (R^2^_C_) and prediction (R^2^_P_) were >0.82 and >0.86, with RPD values of >2.20 for 10 amino acids, resulting in a high prediction accuracy. The PLS models for glutamic acid, leucine, lysine and valine have to be improved. The prediction of six fatty acids was also possible with the coefficient of the determination of calibration (R^2^_C_) and prediction (R^2^_P_) > 0.77 and >0.66 with RPD values > 1.73. Only the prediction accuracy of palmitic acid was very weak, which was probably due to the narrow variation range. NIRS could help insect producers to analyze the nutritional composition of *Tenebrio molitor* larvae fast and easily in order to improve the larval feeding and composition for industrial mass rearing.

## 1. Introduction

The growing world population will reach over nine billion people by 2050 [1], which means the current food production will have to be doubled [2]. Environmental degradation and climate change are reducing the areas worldwide available for food production as well as having a negative impact on food and feed productivity, making it necessary to search for sources of protein other than fish and soybean for livestock feeding [3,4]. Insects are a major focus of attention as an alternative protein source [5], especially the black soldier fly (*Hermetia illucens* L., Diptera: Stratiomydae), yellow mealworm (*Tenebrio molitor* L., Coleoptera: Tenebrionidae) and locusts. All are well-studied and promising edible insects for industrial feed production [3]. The yellow mealworm is found worldwide and is a general storage pest [6]. As a holometabolous insect, the mealworm goes through several different life stages (egg, larva, pupa, beetle) where the morphology and composition vary greatly [7,8]. There is also variability in the development time [7,9], as the number of larval stages can vary from 9 to 24, depending on various influencing factors such as temperature, humidity, oxygen concentration, density, and substrate quality and composition [8,10,11,12,13,14]. *Tenebrio molitor* larvae show a high water, protein and fat content as well as vitamins (e.g., vitamin B_1_, B_2_, B_12_) and minerals (e.g., magnesium, zinc) [2,4]. The protein content has a high variance between 25 and 75% on a dry matter basis [15,16], and the fat content, too, can range between 10 and 50% (dry matter basis) [17,18,19]. The yellow mealworm contains all the essential amino acids needed for human nutrition [16,20] and also has a high amount of unsaturated fatty acids [20,21]. The protein and lipid accumulation as well as the amino and fatty acid profile in insects depends primarily on diet, developmental stage and species as well as environmental conditions (e.g., temperature and humidity) [21,22,23]. In general, mealworm larvae contain large amounts of oleic acid, linoleic acid and palmitic acid, which may be a result of their diet consisting usually of grains and by-products with high oleic acid, linoleic acid, and palmitic acid contents [21,23]. The amino acid composition of *Tenebrio molitor* larvae is highly variable, but some amino acids, including alanine, aspartic acid, glycine and tyrosine are often the most abundant amino acids, while histidine, cysteine and methionine are the least abundant [15,16,20].

However, there is still a lack of knowledge regarding the different instars and the nutritional composition of *Tenebrio molitor* larvae. In industrial rearing, larvae are usually harvested when the first pupae are visible. This point in time is often too late, since valuable nutrients such as fatty acids are on the decline, as insects prepare for energy-intensive metamorphosis and use fat, for instance, as a source of power [24]. Therefore, it is important to know the nutritional composition of the different instars of *Tenebrio molitor* in order to determine whether harvesting at an earlier larval instar is more appropriate.

The supplementation of essential amino acids is considered important for livestock, such as pigs, chicken or aquaculture fish, to satisfy the requirements of these animals and to increase growth performance and reproductive efficiency as well to improve feed consumption and digestion [25,26,27]. The best way to cover the amino acid requirements of livestock is to feed diets composed of a mixture of ingredients with an optimal nutritional composition that provides the desired amino acid balance. Our previous study revealed that it is possible to influence the nutrient composition of mealworm larvae by giving them appropriate feed [28,29]. Therefore, it could be an option to influence the composition of *T. molitor* larvae so that the nutrient composition for livestock feeding will be considered optimal, meaning that no further supplementation is needed. It is essential to know the amounts of amino and fatty acids in order to calculate the suitable inclusion level in a complete insect diet and to change the insect composition.

Consequently, a fast and non-destructive method is needed for determining amino and fatty acids, which led to the use of near-infrared reflectance spectroscopy (NIRS) in this study. NIRS is an analytical method that has many advantages: it is a fast technique with results in less than one minute, requires no sample preparation (liquids and solids can be used in pure form) and no waste is generated, making it an environmentally friendly method. Spectroscopic analysis with chemometric methods has proved to be effective in determining the amino acid and fatty acid composition in food, feed and pharmaceuticals [30,31,32]. The successful use of NIRS for the analysis of water, protein, and fat in different food products, such as cereals [33,34], cheese [35,36], fruit [37] and meat [38,39,40] has been established. Some spectroscopic applications that could be used for the detection of insect pests in stored grains are also known [41,42], but those applications could not be used for analyzing the insect chemical composition. The detection of the protein content of insect-based energy bars [43] and the classification of edible insect powders using Fourier-transform infrared spectroscopy (FTIR) [44] is also possible. In our previous study, we were able to prove that it is possible to determine the moisture and protein content [29] as well as the fatty acid content [23] of living mealworm larvae using near-infrared reflectance spectroscopy. However, we also wanted to find out whether it is possible to determine amino acid composition of living mealworm larvae with NIRS and to improve the prediction models for the fatty acids, such as palmitoleic and stearic acid, which have insufficient prediction accuracy.

The aims of this study were: (1) to determine the composition of the different larval instars of *Tenebrio molitor* in order to detect possible changes, (2) to search for the suitable point of harvest for industrial mass rearing of mealworm larvae and (3) to obtain NIR calibrations and multivariate classification models to predict the amino and fatty acid content in living mealworm larvae. There is currently no known application for determining the amino acid content of living insect larvae by near-infrared reflectance spectroscopy. Our previous study already focused on detecting fatty acids using NIRS [23], but we did not manage to obtain sufficient prediction models for stearic and linoleic acid, which we wanted to revise in this publication.

This study can help to build an automatic system, based on the calibration data, for predicting the amino acid content in living mealworm larvae in a fast and non-destructive way, allowing for modification in larvae composition during insect rearing.

## 2. Materials and Methods

For creating a calibration and prediction model for the amino and fatty acid content of living mealworm larvae, it is important to collect the NIR data (spectra) and the reference data (amino and fatty acid content). Therefore, we used pre-column derivatization with o-phthalaldehyde (OPA) in combination with high performance liquid chromatography (HPLC) to analyze the amino acid content. Reference data for fatty acids were collected by performing lipid extraction followed by derivatization with sulfuric acid, methanol and n-hexane in combination with gas chromatography (GC). The steps of the method implementation, recording NIR spectra, as well as the amino and fatty acid content, are described below.

### 2.1. Insect Samples

*Tenebrio molitor* larvae used in this study were reared at the University of Applied Sciences Bremerhaven in a stainless-steel container (53.0 long × 32.5 wide × 10.0 cm deep) under controlled conditions at 26 °C with 60–70% relative humidity and were fed with wheat bran (Roland Mills United GmbH & Co. KG, Bremen, Germany) ad libitum. Pure water was given to the larvae daily and evenly distributed in the rearing container with a spray bottle. Larvae from the fifth to the last instar were collected, weighed (ADB 200-4, Kern & Sohn, Balingen-Frommern, Germany), starved for 24 h and measured using NIRS. After recording the data, larvae were frozen at −21 °C for 48 h in a freezer (HAS 47520, Beko, Neu-Isenburg, Germany) and stored prior to performing chemical analyses.

### 2.2. Moisture and Protein Analysis

The moisture and protein content of *Tenebrio molitor* larvae were determined as described previously [29]. The larvae were dried at 103 °C for 4 h in a drying oven (U10, Memmert, Schwabach, Germany) to determine the moisture content. Protein content (% on a fresh weight basis) was estimated by multiplying the measured nitrogen content with a factor of 6.25 using the Kjeldahl method (BVL L 06.00-7:2014-08) [45].

### 2.3. Amino Acid Analysis

The determination of amino acids was based on the Roth method (1971) [46] with some modifications. 

#### 2.3.1. Sample Preparation

First, the lyophilized crude samples were homogenated in a mortar, and to 10.0–10.5 mg of the samples, 2 mL H_2_O (ultrapure, Purelab Flex 2, Elga LabWater, Celle, Germany) and 2 mL hydrochloric acid (37%, fuming, Carl Roth GmbH, Karlsruhe, Germany) were added. After gassing with nitrogen, hydrolysis took place in sealed hydrolysis vials (Schott, Mainz, Germany) in a heating oven (110 °C, 20 h, Heraeus, Hanau, Germany). After hydrolysis, the samples were adjusted to a pH of 8.5 with the addition of a boric acid sodium hydroxide solution (pH 14, Carl Roth GmbH, Karlsruhe, Germany, ice cooling) and diluted to 25 mL with H_2_O (ultrapure). An amount of 1 mL of the hydrolysate was centrifuged (15,000 rpm, 5 min, Universal 320 R, Hettich, Tuttlingen, Germany). The supernatant was diluted 1:40 with H_2_O (ultrapure) filtered (0.20 µm; Rotilabo, Carl Roth GmbH, Karlsruhe, Germany) and stored at 4 °C until further use.

#### 2.3.2. Derivatization

The derivatization was carried out as pre-column derivatization with OPA-reagents (Phthalaldehyde Reagent, Sigma-Aldrich, Taufkirchen, Germany) according to a standard laboratory protocol. In brief, 50 µL of the sample were mixed with 14 µL of OPA reagent and incubated (3 min, room temperature) in a HPLC vial.

#### 2.3.3. HPLC Analysis

The HPLC analysis was performed using a Shimadzu LC 10 system equipped with a fluorescence detector (RF-10A, Shimadzu, Duisburg, Germany). The samples were separated on a Kinetex C-18 HPLC column (2.6 µm; 150 × 4.6 mm with guard; Phenomenex LTD, Aschaffenburg, Germany) at 32 °C using a solvent gradient (Eluent A: sodium acetate (1.64 g/L), pH 6.0 adjusted with acetic acid (10%)); Eluent B: sodium acetate (1.64 g/L), 400 mL acetonitrile, 400 mL methanol, 21 mL acetic acid (10%)) with Eluent A at 90% to 60% in 40 min to 0% in 25 min, constant for another 3 min, back to 90% in 7 min and constant for another 10 min. The flow rate was held at 0.4 mL/min. For each analysis, an aliquot of 10 µL of sample was injected. Detection was performed using an excitation wavelength of 330 nm and an emission wavelength of 450 nm using Clarity (DataApex, Prague, Czech Republic) for data acquisition, data processing and instrument control.

The amino acid composition was identified and quantified by comparison with an amino acid analytical standard (AAS18, Sigma Aldrich, Taufkirchen, Germany). For this purpose, 3µL of the standard was injected daily, using the solvent gradient described above. It should be emphasized that AAS18 contains only 17 amino acids. Glutamine and asparagine are not included, as these are detected in total as aspartate and glutamate. Cysteine is only present as the dimer cystine and tryptophan is also missing.

### 2.4. Lipid Content and Fatty Acid Analysis

#### 2.4.1. Lipid Extraction

For lipid extraction in the ultrasonic bath (10 min, Sonorex, Bandelin electronic GmbH, Berlin, Germany), 25 mg of the sample material was mixed with 6 mL DCM (Carl Roth GmbH, Karlsruhe, Germany) in methanol (2:1, *v*/*v*, Carl Roth GmbH, Karlsruhe, Germany) and (if the sample is used for derivatization (2.4.2)) with 100 µL internal standard (FAME 23; Carl Roth GmbH, Karlsruhe, Germany). The liquid phase is removed and the remaining sample material is re-extracted twice more, each time with 3 mL DCM in methanol (2:1, *v*/*v*) for 5 min. The extracts are combined in the separatory funnel and 5 mL 0.88% KCl solution and 5 mL DCM are added and shaken for 3 min (pressure equalization). The nonpolar phase is transferred to a flask and the aqueous phase is re-extracted twice with 5 mL DCM. The nonpolar extracts are combined and evaporated to dryness in a rotary evaporator (water bath 30 °C, 630 mbar, 120 rpm, Rotavopor R300, Büchi, Essen, Germany). The extraction residue is resuspended in 1 mL DCM and quantitatively transferred to a Schott glass. The DCM is subsequently evaporated with nitrogen and the lipid content of the extract sample was determined.

#### 2.4.2. Derivatization

An amount of 2 mL of the derivatization reagent as stock solution of 1.5 mL sulfuric acid (37%, Carl Roth GmbH, Karlsruhe, Germany) in 50 mL Methanol and 500 µL of n-hexane (Carl Roth GmbH, Karlsruhe, Germany) was added to the extract and esterified for 4 h at 80 °C in a thermoblock (analog heatblock, VWR, Darmstadt, Germany). After derivatization, a further 4 mL of ultrapure H_2_O and 3 mL of n-hexane were added in order to extract the fatty acid methylic esters (repeated three times). The organic layers are combined and then evaporated to a small residual volume in a rotary evaporator (50 °C, 350 mbar, 120 rpm). The remainder was transferred to GC vials and evaporated to dryness with nitrogen. Subsequently, the dry weight of the extract was determined. For re-suspension, 1 mL n-hexane was added and the sample vortexed. Finally, the sample was stored at 4 °C until further use.

#### 2.4.3. Gas Chromatography

The measurement was performed on an HP Agilent 6890 (G1530A) Plus Gas Chromatograph equipped with a PAL autosampler and FID detector (Agilent, Waldbronn, Germany). The carrier gas was helium (1.2 mL/min) and the fatty acids were separated on a Trace TR-FFAP column (length: 30 m, diameter: 0.25 mm, film thickness: 0.25 µm; Thermo Fisher Scientific, Darmstadt, Germany). The gas flows for FID were hydrogen 35 mL/min and compressed air 350 mL/min. The injector/detector temperatures were 240 °C and 280 °C, respectively. Split injection with split ratio of 25:1 was performed with 1 µL of the re-suspended sample. The oven temperature program was T1 60 °C /start, T2 200 °C (heating rate 15 °C/min), T3 240 °C (heating rate 5 °C/min; hold for 10 min) and the cool down by 60 °C/min to 60 °C. Fatty acids were identified by comparison to a fatty acid standard-mix (Supelco^®^ 37 Component FAME Mix, Sigma-Aldrich, Taufkirchen, Germany) using Clarity (Version 8.8, DataApex, Prague, Czech Republic) for data acquisition, data processing and instrument control.

### 2.5. Spectra Collection

A glass petri dish (internal diameter: 70 mm, depth: 20 mm) was used to measure living mealworm larvae. An infrared reflectance spectrometer (PSS 2120, Polytec GmbH, Waldbronn, Germany) was used to collect the spectral data at a wavelength between 1100 and 2100 nm. Spectra from all samples were measured ten times and averaged out of 50 spectra.

### 2.6. Multivariate Analysis

NIR spectral data were processed using Matlab (version R2020a, The MathWorks Inc., Natick, MA, USA) and PLS Toolbox (version 8.9.1, Eigenvector Research Inc., Wenatchee, WA, USA) to develop a calibration model for predicting the amino acid and fatty acid content of living *Tenebrio molitor* larvae. Samples were randomly partitioned into two subsets, a calibration set (*n* = 80) and an independent validation set (*n* = 40), to evolve the prediction equations. As the most commercially multivariate analysis method, partial least squares (PLS) regression was used to receive classification models in order to distinguish the differences in amino acid and fatty acid content. In previous studies, PLS regression is deemed to perform best among different models using NIR raw spectra [47]. Preprocessing was performed using standard normal variates (SNV) and 1st (1D) order derivatives. The maximum variance was used to determine the optimal number of latent variables (LV). A number of parameters were used to evaluate the predictive capability and accuracy of the PLS models, including coefficient of determination for calibration (R^2^_C_; Equation (1)), coefficient of determination for prediction (R^2^_P_; Equation (2)), root mean square error of calibration (RMSEC; Equation (3)) and root mean square error of prediction (RMSEP; Equation (4)) and were calculated as follows:

(1) $${R^{2}}_{C}=\frac{\sum_{i=1}^{n_{c}}{\left({\hat{y}}_{i}-y\right)}^{2}}{\sum_{i=1}^{n_{c}}{\left(y_{i}-y\right)}^{2}}$$

(2) $${R^{2}}_{P}=\frac{\sum_{i=1}^{n_{p}}{\left({\hat{y}}_{i}-y\right)}^{2}}{\sum_{i=1}^{n_{p}}{\left(y_{i}-y\right)}^{2}}$$

(3) $$RMSEC=\sqrt{\frac{1}{n_{c}}\sum_{i=1}^{n_{c}}{\left({\hat{y}}_{i}-y_{i}\right)}^{2}}$$

(4) $$RMSEP=\sqrt{\frac{1}{n_{p}}\sum_{i=1}^{n_{p}}{\left({\hat{y}}_{i}-y_{i}\right)}^{2}}$$

where

y = indicates data to be fitted with a mean value;
${\hat{y}}_{i}$ = predicted value of the *i*th observation;
$y_{i}$ = measured value of *i*th observation;
*n*_c_ = number of observations in calibration set;
*n*_p_ = number of observations in prediction set.

In order to determine the practical applicability of the prediction models, the ratio of performance to deviation (RPD; Equation (5)) was calculated as follows:

(5) $$RPD=\frac{SD}{RMSEP}$$

where

SD = prediction set standard deviation.

The RPD values must be greater than 10 in order to be equivalent to the reference method, above 3 for routine analysis, and over 2 for a robust calibration. There is no predictive power in models with an RPD value less than 1.5 [48,49]. We chose the models that had the highest R^2^_p_ and RPD, the lowest RMSEP values and a limited number of latent variables (≤10).

### 2.7. Statistical Analysis

In order to determine the significance of the results, three replicates (*n* = 3) were conducted. SigmaPlot 12.5 (Systat Software Inc., Düsseldorf, Germany) were used to check for normality (Shapiro–Wilk test) and homogeneity of variances (Bartlett’s test). The data was analyzed using one-way ANOVA and Tukey–Kramer post-hoc tests with a 95% confidence interval (*p* < 0.05).

## 3. Results

### 3.1. Growth Parameter

**Figure 1:**
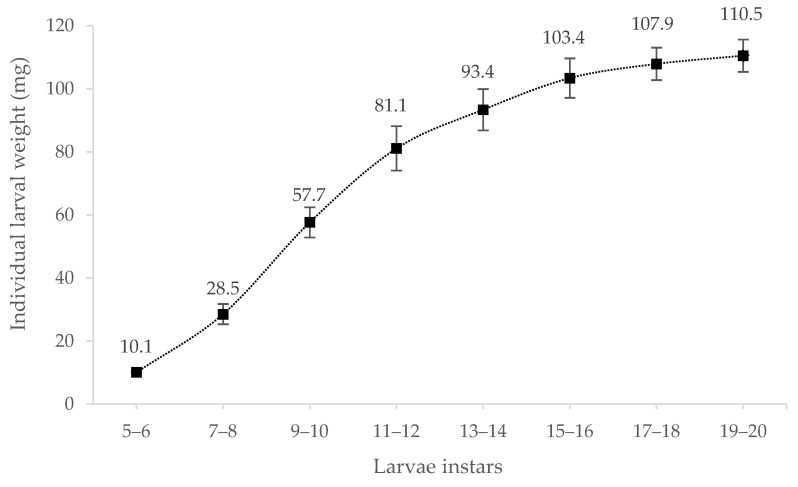
Individual larval weight (mg) of *Tenebrio molitor* larvae in different instars (5 to 20, *n* = 100).

In this experiment, *Tenebrio molitor* larvae had 20 instars before the first pupae appeared. The individual larval weight increased with larval development (Figure 1). In addition, the standard deviation increased in later growing instars, suggesting that larvae grew more inhomogeneously at a later instar stage.

### 3.2. Proximate Analysis

The proximate analysis on a fresh weight basis of *Tenebrio molitor* larvae of different larval instars (5 to 20) is presented in Table 1. The main components of the larvae are water, protein and fat. The moisture content decreased slightly from 67.3 ± 0.2% with growing larvae and reached its minimum with 57.7 ± 1.5% in the final instars. The percentage of protein in fresh weight decreased from 25.7 ± 0.6% (instar 5–6) to 21.3 ± 0.2% (instar 19–20) with increasing development. From larval instar 11 to 18 the protein content remained almost constant. The fat content increased from 4.9 ± 0.0% in the early instar (5–6) to 15.7 ± 0.7% as the larval development progresses to instar 19–20.

### 3.3. NIR Spectra

**Figure 2:**
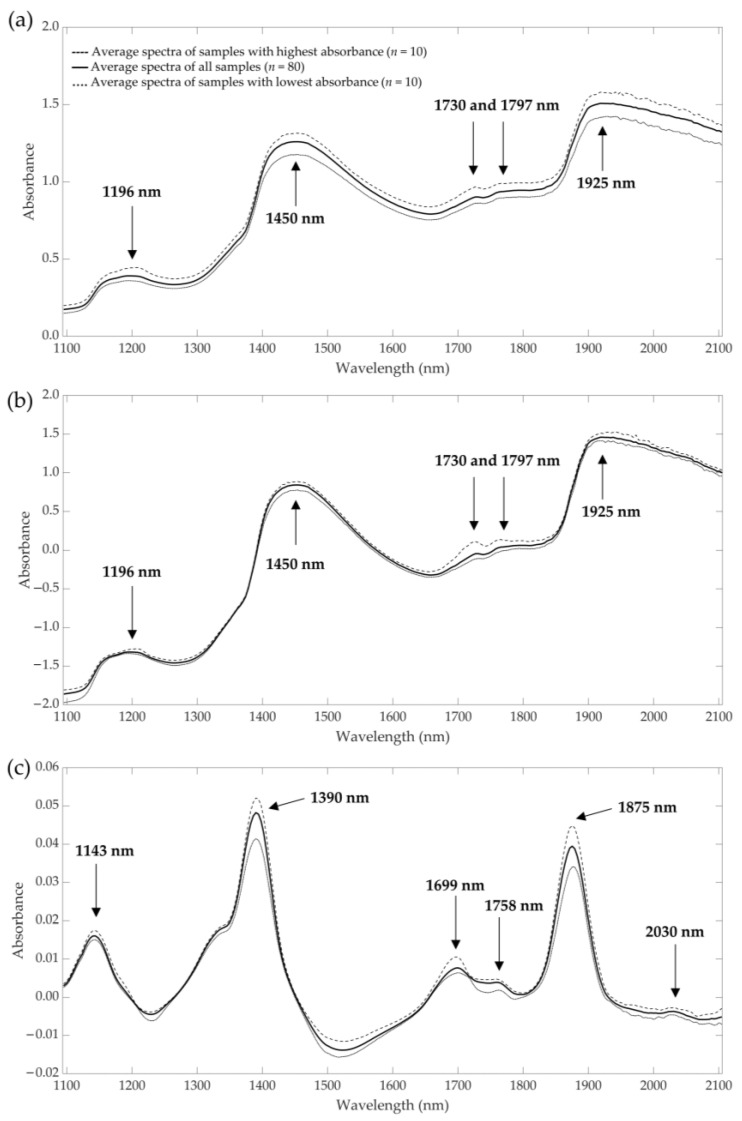
Average NIR raw spectra (**a**) and preprocessed spectra with standard normal variate (**b**) and first derivative (**c**) of living *Tenebrio molitor* larvae from samples with the highest and lowest absorbance compared to all samples (mean, *n* = 80).

The average near-infrared raw spectra and preprocessed data (standard normal variate and first derivative) are presented in Figure 2. Further details of the NIR raw and preprocessed data of all groups can be found in Supplementary Materials (Figure S1). In the raw spectra (Figure 2a), five different peaks can be identified at wavelengths of about 1196, 1450, 1730, 1797 and 1925 nm. The peaks in the preprocessed data with standard normal variate were the same as in the raw spectra, but the absorbances with the first derivative differs. Peaks at wavelengths of 1143, 1390, 1699, 1758, 1875 and 2030 nm could be identified. Compared to the raw spectra, pretreated spectra with first derivative spectra showed more information, and where other studies were presented using derivative spectra for prediction, there was better accuracy than when using raw spectra [50]. Therefore, first derivative and standard normal derivative were used in this research study to predict amino and fatty acid content.

### 3.4. Prediction of Amino Acid Content

**Figure 3:**
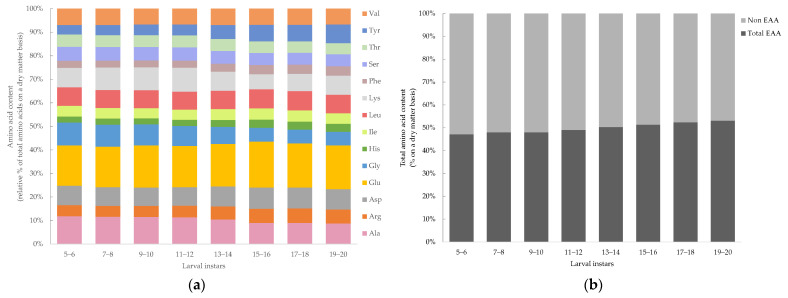
(**a**) Amino acid content (%) and (**b**) essential and non-essential amino acid content (%) of *Tenebrio molitor* larvae of different larval instars (5 to 20) on a dry matter (DM) basis analyzed by HPLC with OPA derivatization. Data are presented as mean, *n* = 4.

The amino acid content of *Tenebrio molitor* larvae of different larval instars (5–20) are presented in Figure 3a. The mean and standard deviation with significant differences of the amino acid contents are provided in the Supplementary Material (Table S1). The following amino acids were detected, including alanine (Ala), arginine (Arg), aspartic acid (Asp), glutamic acid (Glu), glycine (Gly), serine (Ser), tyrosine (Tyr), histidine (His), lysine (Lys), threonine (Thr), isoleucine (Ile), leucine (Leu), phenylalanine (Phe) and valine (Val). The levels of tryptophan and sulfur amino acids (cysteine and methionine) are not presented, as they were lost during acidic hydrolysis, and proline which contains a secondary amino group is not detectable by the OPA-method [51]. Significant variations among the larval instar were observed. Alanine, glycine and serine decreased continuously and had the lowest content at larval instar 19–20 (Ala: 8.74 ± 0.19%; Gly: 5.77 ± 0.14%; Ser: 5.14 ± 0.07%). Tyrosine and phenylalanine increased with larval development and had the highest content at instar 19–20 (Tyr: 7.98 ± 0.12%; Phe: 4.02 ± 0.06%). For leucine, valine and isoleucine, no significant differences in relation to the larval instar could be determined. The instar 5–6 had the highest content of arginine (11.82 ± 0.17%), glycine (9.69 ± 0.10%), serine (6.00 ± 0.04%), threonine (5.14 ± 0.00%) and valine (6.95 ± 0.05%). Lysine increased during larval growth, reaching a peak at instar 11–12 (10.23 ± 0.01%), and then decreased to 8.09 ± 0.96%, which was comparable to the initial level at instar 5–6 (8.27 ± 0.63%). All essential amino acids (His, Ile, Leu, Lys, Phe, Thr, Val and Arg) were added to determine the total essential amino acid content of mealworm larvae of different instars. The content of essential amino acids, presented in Figure 3b, showed slightly (47.12–53.13%) but significant (*p* < 0.05) differences between the larval instars, whereas the essential amino acid content increased with larval development. The means and standard deviations with significances are provided in the Supplementary Material (Table S1).

Table 2 presents the descriptive statistics for the amino acid content of the living *Tenebrio molitor* larvae in the calibration and validation sets. The data describe a wide range of variability in some amino acids, such as alanine (8.74 to 11.82%), glycine (5.77 to 9.69%) and tyrosine (4.02 to 7.98%), which is beneficial for calibration development and prediction accuracy [40]. This variability of the other amino acids was lower, suggesting that the influence of the larval instar do not have such a large impact on arginine, aspartic acid, glutamic acid, histidine, isoleucine, leucine, lysine, phenylalanine, serine, threonine and valine. During the development of the prediction equality, no outliers were detected.

Table 3 summarizes the performance of the PLS models for the amino acid content in *Tenebrio molitor* larvae in the calibration and validation sets, which is based on the NIR raw spectra using mathematical preprocessing treatments. Two methods, standard normal variate (SNV) and the first derivative (1D), were considered as primary pretreatments for spectral data. An optimal combination of algorithm parameters, such as window size, polynomial order and derivative order (first or second), was established on the basis of standard error of cross-validation of PLS calibrations. The best results were obtained with standard normal variate for most of the amino acid spectra except for glutamic acid, leucine and lysine, which required first-order derivation. Only phenylalanine did not require preprocessing to get the best results.

**Figure 4:**
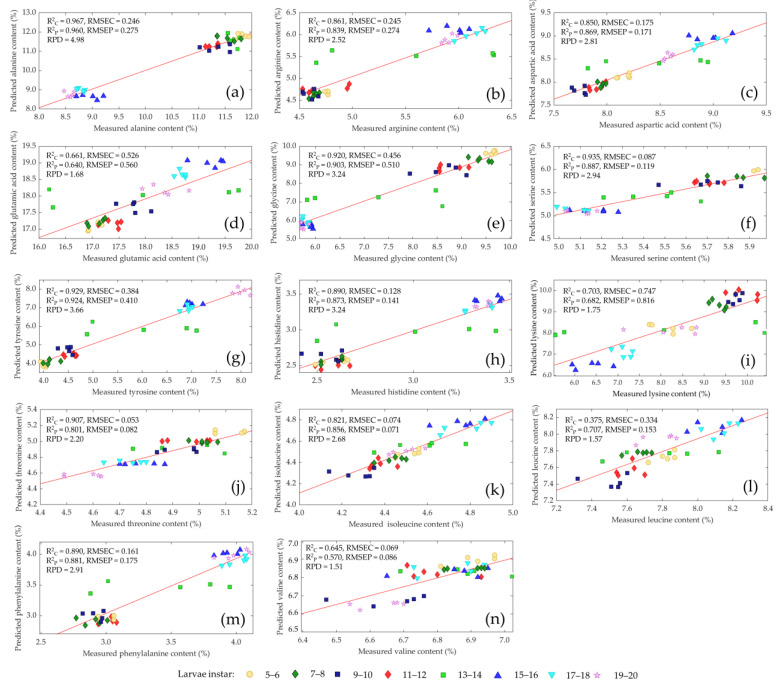
Comparison of measured and predicted values of alanine (**a**), arginine (**b**), aspartic acid (**c**), glutamic acid (**d**), glycine (**e**), serine (**f**), tyrosine, (**g**), histidine (**h**), lysine (**i**), threonine (**j**), isoleucine (**k**), leucine (**l**), phenylalanine (**m**) and valine (**n**) of living mealworm larvae with different larvae instars (• 5–6, ♦ 7–8, ■ 9–10, ♦ 11–12, ■ 13–14, ▲ 15–16, ▼ 17–18, ★ 19–20) in validation sets using near-infrared spectroscopy.

Figure 4 shows the comparison of measured and predicted values of the amino acids of living mealworm larvae with different instars in validation sets. The correlation coefficient for determining calibration (R^2^_C_) was >0.92 and (R^2^_P_) > 0.90 for prediction. RPD values were >3.20 for alanine, glycine and tyrosine, and this is regarded as adequate for routine analysis for the three amino acids. Serine and threonine reached an R^2^_C_ > 0.90 and R^2^_P_ > 0.80 with RPD values > 2.20, which indicated a robust calibration for the two amino acids [48,49] The RPD values for arginine, aspartic acid, histidine, isoleucine, serine, threonine and phenylalanine ranged from 2.20 to 2.91 with R^2^_C_ and R^2^_P_ > 0.80 and represented a good calibration. Approximate quantitative predictions and the ability to differentiate between low and high values were obtained for glutamic acid, leucine, lysine and valine with RPD values > 1.51 and R^2^_C_ > 0.64 and R^2^_P_ > 0.57.

### 3.5. Prediction of Fatty Acid Content

**Figure 5:**
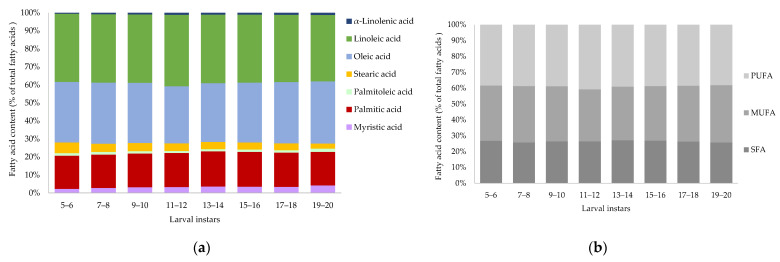
(**a**) Fatty acid content (relative % of total fatty acids) and (**b**) saturated (SFA), monounsaturated (MUFA) and polyunsaturated (PUFA) fatty acids (relative % of total fatty acids) of *Tenebrio molitor* larvae of different larval instars (5 to 20) on a dry matter (DM) basis analyzed by GC-FID. Data are presented as mean, *n* = 4.

The fatty acid composition (relative % of total fatty acids on a dry matter basis) of *Tenebrio molitor* larvae of different larval instars (5 to 20) is presented in Figure 5a. The mean and standard deviation with significant differences of the fatty acids are described in the Supplementary Material (Table S2). The following fatty acids were detected: myristic acid (C14:0), palmitic acid (C16:0), palmitoleic acid (C16:1), stearic acid (C18:0), oleic acid (C18:1 n-9), linoleic acid (C18:2 n-6) and α-linolenic acid (C18:3 n-3). All larval instars of *Tenebrio molitor* have a high content of polyunsaturated fatty acids (38.14–40.82%) (Figure 5b), whereas α-linolenic acid increased significantly with growing larvae. Stearic acid was highest (6.02 ± 0.07%) in instar 5–6 and decreased to 2.85% at a later larval instar stage, while myristic acid and palmitoleic acid increased significantly, and the peak was reached at 4.16 ± 0.05% for myristic acid and at 1.74 ± 0.05% for palmitoleic acid in the last larval instar. Palmitic acid, oleic acid and linoleic acid did not differ significantly when comparing larval instars.

Table 4 describes the statistics for the fatty acid content of the living mealworm larvae in the calibration and validation sets. The data presented a wide range of variability for the fatty acids, except palmitoleic acid (1.04 to 1.80%) and α-linolenic acid (0.51 to 1.25%). During the development of the prediction equality, no outliers were detected.

Table 5 outlines NIR prediction models for the fatty acid content in *Tenebrio molitor* larvae in the calibration and validation sets, which was based on the NIR raw spectra using different mathematical treatments. As primary pretreatments for the fatty acid spectral data, standard normal variate (SNV) and the first derivative (1D) were considered as suitable. Optimal combination of algorithm parameters was established on the basis of standard error of cross-validation of PLS calibrations. The best results were obtained with standard normal variate for myristic acid, palmitoleic acid and oleic acid. Palmitic acid, linoleic acid and α-linolenic acid required a first-order derivation, while stearic acid needed no mathematical pretreatment to get the best results.

**Figure 6:**
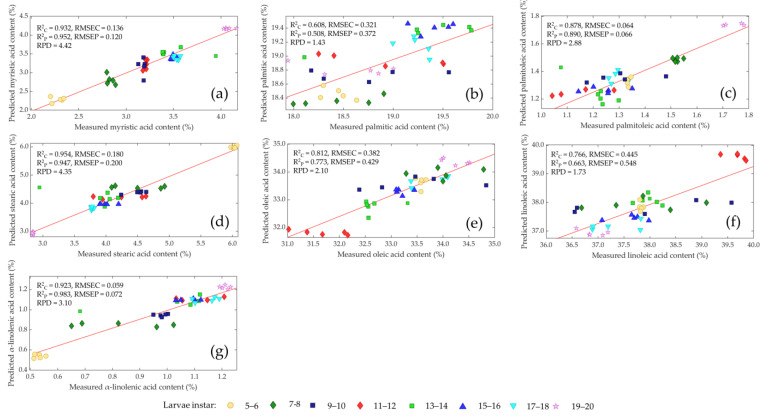
Comparison of measured and predicted values of myristic acid (**a**), palmitic acid (**b**), palmitoleic acid (**c**), stearic acid (**d**), oleic acid (**e**), linoleic acid (**f**) and α-linoleic acid (**g**) of living mealworm larvae with different instars (• 5–6, ♦ 7–8, ■ 9–10, ♦ 11–12, ■ 13–14, ▲ 15–16, ▼ 17–18, ★ 19–20) in validation sets by near-infrared spectroscopy.

The performance of the chosen PLS models with regard to the measured versus predicted values of the different fatty acids are shown in Figure 6. The best model was selected in terms of high RPD and R^2^_P_, a low RMSEP, and the limitation of the number of latent variables (<10). The statistics demonstrate the ability to predict the fatty acid content of living *Tenebrio molitor* larvae. However, a mathematical preprocessing of the raw spectra, except for stearic acid, is essential to increasing the prediction accuracy of the NIR models. The correlation coefficient for determining calibration (R^2^_C_) was >0.90 and prediction (R^2^_P_) > 0.89, and RPD values were >3.10 for myristic acid, stearic acid and α-linolenic acid; this was deemed sufficient for routine analysis of the three fatty acids. Palmitoleic acid and oleic acid achieved an R^2^_C_ >0.81 and R^2^_P_ > 0.77 with RPD values > 2.10, which represents a robust calibration for the two fatty acids. Approximate quantitative predictions and the possibility to make distinctions between low and high values were obtained for linolenic acid with R^2^_C_ > 0.76, R^2^_P_ > 0.66 and RPD value > 1.73. The prediction of palmitic acid was unsuccessful, and the PLS model had no prediction ability as R^2^_C_ = 0.61, R^2^_P_ = 0.51 and RPD value = 1.43.

## 4. Discussion

In our study, mealworm larvae had up to 20 larval instars, which is within the range of 9–24 instars reported by other authors [11,12,14]. The factors that cause the plasticity of the instar number are multiple. Environmental factors such as temperature, humidity, food quality and quantity, photoperiod and rearing density affect the number of insect instars [9]. There are several insect species that have an increased instar number as a result of low temperature and humidity as well as low quality and quantity of food (transient starvation). Our larvae were fed ad libitum with wheat bran and thus an undersupply of feed can be excluded. However, there is the possibility of influencing growth by adding further substrates like additional protein sources (brewer’s yeast, pea or rice protein, etc.) so that faster growth rates may lead to a lower number of larval stages. Additionally, the water source and amount can have a significant influence on the growth rate, as we showed in our previous study [29]. We provided pure water to the larvae. However, an increasing amount of water could possibly result in faster development. In our insect rearing room, we had a temperature of 26 °C and a relative humidity of 60–70%, which is according to the literature within the optimum range of the larvae [14,52]. Nevertheless, some studies showed that higher temperature (27–29 °C) and humidity (75–80%) can affect larval growth and lead to faster growth rates [14,29,53]. As a result of the lower temperature and humidity, our larvae may have grown more slowly and had, as a consequence, more larval stages.

The moisture, protein and fat content of *Tenebrio molitor* larvae of this study are comparable to the data reported by other studies [17,19,52,54,55]. However, protein content is usually calculated by multiplying nitrogen content with a protein factor of 6.25 and is therefore normally overestimated due to the presence of non-protein nitrogen from chitin [56]. In this research study, we did not analyze the chitin content of mealworm larvae and deducted that from the protein content or the NIR spectra, so chitin and other nitrogen-containing compounds are included too. This may also have resulted in differences of protein, as chitin content varies by larval stage, which was reported by Toviho and Bársony (2022) [57].

Our research demonstrates that the nutrient content is affected by larval instar, which was also observed in similar investigations [8,57,58]. Normally, the final instar has the highest lipid stores [59] and increases with development but decreases significantly in the adult, since fat is needed as an energy source during pupation [60]. But not only the varying instars have an influence on the nutritional composition of the larvae; other factors can also have a major impact. Some previous examinations showed that there are variations in the nutrient composition between different and similar insect species as a result of different diets and feeding [61,62]. Environmental factors like temperature, humidity and light can also affect the development, growth and nutrient composition of insects [53,63]. Linoleic and oleic acids are the major unsaturated fatty acids of *Tenebrio molitor* larvae in this study, which coincides with the results of other studies [20,64]. The fatty acid composition of the larvae is mostly influenced by the diet, temperature, life stage, species and sex, as males of most insects have a lower fat content than females [65,66,67]. Commercially raised insects generally contain a large amount of oleic and linoleic acid because their diet often contains a high content of grains and grain by-products [22,56].

As the first research study to predict amino acid content in living insect larvae using near-infrared reflectance spectroscopy, the performance of the models was comparable or better than soy [68,69], fishmeal [70] and Chinese rice wine [71]. The measurement of living mealworm larvae using NIRS is very difficult because of their structure as a complex matrix. The nutrient content can be predicted more accurately in homogenized samples such as insect flour [72] as compared to intact samples [73]; nevertheless, it is still possible. However, it is easier to create prediction models for macronutrients (e.g., crude protein and crude fat) than for micronutrients (e.g., amino and fatty acids), as demands on measurement technology increase and as we can see in this study compared to our previous research [23,29]. Most relevant spectra peaks were observed between 1390 and 1450 nm (combination C-H stretching and first overtone O-H stretching) and around 1925 and 2030 nm (combination and first overtone O-H and N-H stretching), which is mainly related to the water and protein content [74], around 1143 and 1196 nm (second overtone C-H stretching) and between 1699 and 1797 nm (first overtone C-H stretching), which is generally associated with the fat and fatty acid content [74,75,76]. It was not possible to determine methionine, proline, asparagine, glutamine and tryptophan in mealworm larvae. Reference values based on chemical determination methods are required for the development of the NIR prediction models for amino acids. In this study, HCl hydrolysis and subsequent HPLC analysis after OPA derivatization was used for measuring the amino acids. This reaction causes up to 65% degradation of several amino acids [77]. In addition, amino acids containing sulfur, such as methionine, are more sensitive and degrade quickly in HCl during the sample processing [70,78]. Therefore, no predictive models could be constructed for the missing amino acids using NIRS. Difficulties in quantifying and detecting some amino acids using NIRS in rice and animal feed were also found in other studies [79,80]. The calibration for the amino acid glutamic acid, leucine, lysine and valine showed a need for improvement in the precision of the models. The results demonstrate that the prediction of amino acids depends on the specificity of the amino acids. Low prediction accuracy might relate to the less accurate reference values due to the minor response of these amino acids. However, the preparation of the larvae can have an influence on the composition too. To perform the chemical analysis of the larvae, it is necessary to deactivate them. Research on the Black Soldier Fly (*Hermetia illucens*) showed that the killing method affects the nutritional content and the quality of proteins [81]. According to some studies, slow killing of *H. illucens* by freezing activates several metabolic pathways (melanization, energy metabolism, lipolysis), which includes the consumption and loss of some amino acids (e.g., tyrosine, cysteine, lysine) [81] as well as a relevant release of some fatty acids [82]. Therefore, it is possible that this may also be an explanation for the weak correlation of some amino and fatty acids.

The amino and fatty acid content and composition of the larvae in our study met the recommendations for human requirements [83]. Four amino acids are most likely to be limiting, including lysine, methionine, tryptophan and threonine [83]. The World Health Organization (WHO) recommends an intake of 30–45 mg/kg per day of lysine and of 10–20 mg/kg per day of threonine for adult humans [83]. Our larvae met these requirements with a lysine content ranging between 6.4 and 10.2 g/100 g protein on a dry matter basis and a threonine content of 4.6–5.1 g/100 g protein on a dry matter basis. Consequently, as a diet supplement, mealworm larvae can increase the intake of essential amino acids for adults. The content of essential amino acids increased in growing larvae, which makes the use of older larvae more attractive. However, lysine and threonine content decrease with increasing larval development. The levels of methionine and tryptophan were not determined in this research due to limited detectability with the OPA method used. To overcome this limitation, an alternative approach by Kambhampati et al. (2019) using LC-MS will be of interest [84]. In contrast to the OPA method, however, this approach is not yet a standard assay in amino acid analysis and has therefore not been used to date. Normally, the content of methionine (0.6–1.0 g/100 g protein) and tryptophan (0.3–0.6 g/100 g protein) were reported as low [20,58] and decreased as development progressed, suggesting that harvesting the larvae at earlier larval stages is more optimal. 

In the present study, the prediction accuracy for the fatty acid content of living mealworm larvae proved to be very reliable. Only the model for the prediction of the palmitic acid content needs to be improved. The range of the reference data for myristic acid and stearic acid is higher than for other fatty acids, so the prediction accuracy is better for these models too. In our earlier study, we attempted to build predictive models for all detected fatty acids [23]. However, no suitable predictive model for stearic (R^2^_P_ = 0.509, RPD = 1.96) and palmitoleic (R^2^_P_ = 0.345, RPD = 1.57) acids could be developed. In our current research, we were able to improve the prediction accuracy of stearic (R^2^_P_ = 0.947, RPD = 4.35) and palmitoleic acid (R^2^_P_ = 0.890, RPD = 2.88). Nevertheless, the prediction models for palmitic (R^2^_P_ = 0.508, RPD = 1.43) and linoleic (R^2^_P_ = 0.663, RPD = 1.73) acid were clearly worse than in our previous study. This is related to the differences in the calibration and validation sets in both studies. The precision of the prediction could possibly be improved by a larger variation of the amino and fatty acid content within the calibration. Higher model performance is expected for all fatty acids, if more data points are available, as can be seen in other studies [85,86]. Indeed, the accuracy in predicting the amino and fatty acid content is not precise enough when PLS regression is used a prediction model. It may lead to better accuracy when other chemometric instruments and software are used. Hou et al. (2022) [87] found out that when a decision tree and radial artificial neural network for amino acids were used, a high prediction performance could be ensured. However, artificial neural networks (ANN) are generally like black boxes: it is often not possible to track why a network has made a certain decision. Additionally, the decision algorithm is inadequate for applying regression analysis and for the prediction of continuous values [87]. In order to calculate a generally valid and good result, a large example/training dataset is needed [87]. In this study, the datasets are limited and do not have such an extensive range, so the applicability of a decision tree and ANN was not reasonable. If higher prediction accuracy can be reached, a real-time online monitoring of amino and fatty acid content of living mealworm larvae for large scale production could be realized, which would result in a significant advantage in terms of influencing the nutritional composition of the larvae with specific feeding and the desired nutrient profile at harvest.

Knowing the composition of the larvae makes it easier for industrial rearing to determine the proper time to harvest and to ensure a stable product quality. Usually, larvae are harvested when the first pupae become visible. However, this is not the best time for harvesting, as the larvae are preparing for pupation. This process is very energy-intensive, nutrients are needed for metamorphosis and this results in a decreasing protein content [88]. As this research shows, growth is reduced from larval instar 15–16 and increases only slightly with growing larvae. Therefore, the instar 15–16 is the preferred choice for harvesting because of their favorable and balanced nutrient profile, as the content of most amino and fatty acids decreases with growing larvae. An earlier larval instar for harvesting is also reported and recommended for *Tenebrio molitor* larvae by Yu et al. (2021) [58]. Even though some nutrient values are high (e.g., amino and fatty acids), the bioavailability of these nutrients in the animal and human organism should be analyzed in further studies.

## 5. Conclusions

This study shows the diversity in the different larval instars of *Tenebrio molitor* in terms of the nutrient composition. The results indicate that the water and protein content is highest in young larvae and the fat content is lowest. The latter increases during development. The content of essential amino acids increased with larval development, but the content of the most limiting essential amino acids (e.g., threonine and lysine) decreased. Considering yield, growth rate and nutritional value, an earlier larval instar would be a good choice for a harvest. In particular, the decrease in growth is an important economic factor because the cost of rearing (feed, energy, etc.) should not be ignored. However, further studies need to be conducted in order to determine the risk and cost of feeding under different circumstances in order to choose the optimal harvest time for *Tenebrio molitor* larvae. The results obtained in this research suggested that near-infrared reflectance spectroscopy could be used as a tool to determine several amino and fatty acids in living mealworm larvae economically and rapidly. Nevertheless, further development with larger datasets will be required to enhance the robustness and prediction accuracy of the NIR calibration models.

## Figures and Tables

**Table 1:** Nutritional composition of *Tenebrio molitor* larvae of different larval instars (5 to 20) on a fresh weight (FW) basis (%). Data are presented as mean ± standard deviation, * *n* = 6, ** *n* = 4.

Nutrient Component	Larvae Instars							
	5–6	7–8	9–10	11–12	13–14	15–16	17–18	19–20
Moisture (%) *	67.3 ± 0.2 ^a^	65.8 ± 1.5 ^a^	66.1 ± 1.1 ^a^	65.1 ± 2.1 ^a^	65.6 ± 0.4 ^a^	64.4 ± 0.9 ^a^	63.9 ± 0.8 ^a^	57.7 ± 1.5 ^b^
Crude Protein (% FW) *	25.7 ± 0.6 ^a^	23.7 ± 0.5 ^ab^	22.9 ± 0.1 ^ab^	22.6 ± 0.3 ^b^	22.6 ± 0.7 ^b^	22.7 ± 0.4 ^b^	22.7 ± 0.6 ^b^	21.3 ± 0.2 ^b^
Crude Fat (% FW) **	4.9 ± 0.0 ^b^	7.1 ± 1.8 ^b^	7.0 ± 1.8 ^b^	6.7 ± 2.8 ^b^	11.7 ± 1.0 ^a^	12.3 ± 0.7 ^a^	11.8 ± 1.7 ^a^	15.7 ± 0.7 ^a^

^a–b^ Different superscripts in the same row denote significant differences (*p* < 0.05); protein content was calculated with the conversion factor of 6.25.

**Table 2:** Amino acid content of living *Tenebrio molitor* larvae (values given in % of dry weight) for calibration (*n* = 80) and validation sets (*n* = 40) analyzed by HPLC with OPA derivatization.

Amino Acid	Calibration Set (%)				Validation Set (%)			
	Minimum	Maximum	Mean	SD	Minimum	Maximum	Mean	SD
Ala	8.74	11.82	10.36	1.36	8.47	11.95	10.50	1.37
Arg	4.56	6.38	5.27	0.66	4.53	6.33	5.28	0.69
Asp	7.71	9.18	8.30	0.46	7.67	9.18	8.33	0.48
Glu	15.94	19.86	17.95	0.91	16.19	19.75	17.94	0.94
Gly	5.77	9.69	7.62	1.62	5.70	9.69	7.64	1.65
His	2.48	3.40	2.91	0.39	2.42	3.46	2.92	0.40
Ile	4.20	4.84	4.51	0.18	4.14	4.90	4.53	0.19
Leu	7.38	8.20	7.75	0.42	7.32	8.25	7.80	0.24
Lys	5.61	10.46	8.36	1.38	5.55	10.41	8.36	1.43
Phe	2.71	4.12	3.39	0.49	2.77	4.10	3.40	0.51
Ser	5.02	6.00	5.49	0.34	4.99	6.03	5.51	0.35
Thr	4.54	5.12	4.87	0.17	4.49	5.17	4.88	0.18
Tyr	4.02	7.98	5.64	1.45	3.94	8.19	5.63	1.50
Val	6.53	6.98	6.79	0.12	6.47	7.02	6.80	0.13

SD: standard deviation; Ala: alanine; Arg: arginine; Asp: aspartic acid; Glu: glutamic acid; Gly: glycine; His: histidine; Ile: isoleucine; Leu: leucine; Lys: lysine; Phe: phenylalanine; Ser: serine; Thr: threonine; Tyr: tyrosine; Val: valine.

**Table 3:** Statistics of NIR prediction models for amino acid content in living mealworm larvae.

Amino Acid	Mathematical Treatment	No. of Latent Variables	Calibration Set		Validation Set		
			R^2^_c_	RMSEC	R^2^_p_	RMSEP	RPD
Ala	SNV	8	0.967	0.246	0.960	0.275	4.98
Arg	SNV	8	0.861	0.245	0.839	0.274	2.52
Asp	SNV	8	0.850	0.175	0.869	0.171	2.81
Glu	1D	8	0.661	0.526	0.640	0.560	1.68
Gly	SNV	8	0.920	0.456	0.903	0.510	3.24
His	SNV	8	0.890	0.128	0.873	0.141	2.84
Ile	SNV	8	0.821	0.074	0.856	0.071	2.68
Leu	1D	8	0.775	0.334	0.707	0.153	1.57
Lys	1D	8	0.703	0.747	0.682	0.816	1.75
Phe	None	8	0.890	0.161	0.881	0.175	2.91
Ser	SNV	8	0.935	0.087	0.887	0.119	2.94
Thr	SNV	8	0.907	0.053	0.801	0.082	2.20
Tyr	SNV	8	0.929	0.384	0.924	0.410	3.66
Val	SNV	8	0.645	0.069	0.570	0.086	1.51

SNV: standard normal variate; 1D: first derivative; R^2^_c_: coefficient of determination for calibration; RMSEC: root mean square error of calibration; R^2^_p_: coefficient of determination for prediction; RMSEP: root mean square error of prediction; RPD: ratio of performance deviation; Ala: alanine; Arg: arginine; Asp: aspartic acid; Glu: glutamic acid; Gly: glycine; His: histidine; Ile: isoleucine; Leu: leucine; Lys: lysine; Phe: phenylalanine; Ser: serine; Thr: threonine; Tyr: tyrosine; Val: valine.

**Table 4:** Fatty acid content of living *Tenebrio molitor* larvae (relative % of total fatty acids on a dry matter basis) for calibration (*n* = 80) and validation sets (*n* = 40) analyzed by GC-FID.

Fatty Acid	Calibration Set (%)				Validation Set (%)			
	Minimum	Maximum	Mean	SD	Minimum	Maximum	Mean	SD
Myristic acid (C14:0)	2.18	4.22	3.27	0.52	2.18	4.19	3.25	0.53
Palmitic acid (16:0)	17.83	19.79	18.89	0.52	17.94	19.78	18.91	0.53
Palmitoleic acid (C16:1)	1.04	1.80	1.36	0.18	1.04	1.78	1.36	0.19
Stearic acid (C18:0)	2.82	6.07	4.25	0.85	2.83	6.07	4.24	0.87
Oleic acid (C18:1 n-9)	31.01	34.99	33.29	0.89	31.02	34.83	33.32	0.90
α-Linoleic acid (C18:2 n-6)	36.44	39.84	37.95	0.93	36.55	39.85	37.89	0.95
α-Linolenic acid (C18:3 n-3)	0.51	1.25	1.01	0.22	0.51	1.26	1.00	0.22

SD: standard deviation.

**Table 5:** Statistics of NIR prediction models for fatty acid content in living mealworm larvae.

Fatty Acid	Mathematical Treatment	No. of Latent Variables	Calibration Set		Validation Set		
			R^2^_C_	RMSEC	R^2^_P_	RMSEP	RPD
Myristic acid (C14:0)	SNV	8	0.932	0.136	0.952	0.120	4.42
Palmitic acid (C16:0)	1D	8	0.608	0.321	0.508	0.372	1.43
Palmitoleic acid (C16:1)	SNV	8	0.878	0.064	0.890	0.066	2.88
Stearic acid (C18:0)	None	8	0.954	0.180	0.947	0.200	4.35
Oleic acid (C18:1 n-9)	SNV	8	0.812	0.382	0.773	0.429	2.10
Linoleic acid (C18:2 n-6)	1D	8	0.766	0.445	0.663	0.548	1.73
α-Linolenic acid (C18:3 n-3)	1D	8	0.923	0.059	0.893	0.072	3.10

SNV: standard normal variate; 1D: first derivative; R^2^_C_: coefficient of determination for calibration; RMSEC: root mean square error of calibration; R^2^_P_: coefficient of determination for prediction; RMSEP: root mean square error of prediction; RPD: ratio of performance deviation.

## Data Availability

The data presented in this study are available on request from the corresponding author.

## Supplementary Materials

The following supporting information can be downloaded at: https://www.mdpi.com/article/10.3390/insects14040310/s1, Table S1. Amino acid content of *Tenebrio molitor* larvae of different larval instars (5 to 20) on a dry matter (DM) basis (%) analyzed by HPLC with OPA derivatization. Data are presented as mean ± standard deviation, *n* = 4; Table S2. Fatty acid composition of *Tenebrio molitor* larvae of different larval instars (5 to 20) on a dry matter (DM) basis (relative % of total fatty acids) analyzed by GC-FID. Data are presented as mean ± standard deviation, *n* = 4; Figure S1: Mean average NIR raw spectra (a) and preprocessed spectra with standard normal variate (b) and first derivative (c) of living *Tenebrio molitor* larvae with different instars 5 to 20 (*n* = 10).
