# Supplementary material for: Analysis of the Composition of Different Instars of Tenebrio molitor Larvae using Near-Infrared Reflectance Spectroscopy for Prediction of Amino and Fatty Acid Content
Source: Insects. 2023 Mar 23;14(4):310. doi: 10.3390/insects14040310 (PMC10141721; doi:10.3390/insects14040310)
Supplement: Supplementary file 1 [file insects-14-00310-s001.zip › insects-2266882-supplementary.pdf]

Supplementary material

Analysis of the Composition of Different Instars of *Tenebrio molitor* Larvae using Near-Infrared Reflectance Spectroscopy for Prediction of Amino and Fatty Acid Content

Nina Kröncke [1,*], Stefan Wittke [2], Nico Steinmann [2] and Rainer Benning [1]

[1] Institute of Food Technology and Bioprocess Engineering, University of Applied Sciences Bremerhaven, An der Karlstadt 8, 27568 Bremerhaven, Germany; rbenning@hs-bremerhaven.de

[2] Laboratory for (Marine) Biotechnology, University of Applied Sciences Bremerhaven, An der Karlstadt 8, 27568 Bremerhaven, Germany; swittke@hs-bremerhaven.de (S.W.); nsteinmann@studenten.hs-bremerhaven.de (N.S.)

Table S1. Amino acid content of *Tenebrio molitor* larvae of different larval instars (5 to 20) on a dry matter (DM) basis (%) analyzed by HPLC with OPA derivatization. Data are presented as mean ± standard deviation, $n = 4$.

Amino acid	Larvae instars							
(% DM)	5-6	7-8	9-10	11-12	13-14	15-16	17-18	19-20
Ala	11.82 ± 0.17 ^{a}	11.58 ± 0.14 ^{a}	11.54 ± 0.08 ^{a}	11.36 ± 0.01 ^{a}	10.43 ± 1.84 ^{ab}	8.99 ± 0.05 ^{b}	8.97 ± 0.17 ^{b}	8.74 ± 0.19 ^{b}
Arg	4.74 ± 0.03 ^{c}	4.63 ± 0.03 ^{c}	4.67 ± 0.09 ^{c}	4.92 ± 0.03 ^{bc}	5.57 ± 1.12 ^{ab}	6.00 ± 0.17 ^{a}	6.16 ± 0.07 ^{a}	5.95 ± 0.08 ^{a}
Asp	8.20 ± 0.00 ^{b}	7.95 ± 0.04 ^{b}	7.82 ± 0.06 ^{b}	7.92 ± 0.07 ^{b}	8.49 ± 0.69 ^{a}	9.04 ± 0.22 ^{a}	8.90 ± 0.22 ^{a}	8.62 ± 0.04 ^{a}
Glu	17.16 ± 0.06 ^{c}	17.24 ± 0.10 ^{c}	17.94 ± 0.16 ^{b}	17.49 ± 0.11 ^{c}	18.01 ± 2.51 ^{ab}	19.49 ± 0.02 ^{a}	18.76 ± 0.10 ^{b}	18.60 ± 0.60 ^{b}
Gly	9.69 ± 0.10 ^{a}	9.33 ± 0.31 ^{ab}	8.91 ± 0.24 ^{b}	8.51 ± 0.03 ^{c}	7.28 ± 1.88 ^{d}	5.91 ± 0.04 ^{e}	5.85 ± 0.03 ^{e}	5.77 ± 0.14 ^{e}
His	2.59 ± 0.03 ^{b}	2.59 ± 0.01 ^{b}	2.57 ± 0.02 ^{b}	2.57 ± 0.00 ^{b}	2.97 ± 0.58 ^{ab}	3.42 ± 0.01 ^{a}	3.37 ± 0.02 ^{a}	3.38 ± 0.04 ^{a}
Ile	4.53 ± 0.01 ^{a}	4.44 ± 0.03 ^{a}	4.31 ± 0.01 ^{a}	4.34 ± 0.02 ^{a}	4.58 ± 0.20 ^{a}	4.78 ± 0.09 ^{a}	4.80 ± 0.03 ^{a}	4.52 ± 0.03 ^{a}
Leu	7.86 ± 0.05 ^{a}	7.70 ± 0.03 ^{a}	7.57 ± 0.02 ^{a}	7.62 ± 0.01 ^{a}	7.83 ± 0.36 ^{a}	8.14 ± 0.15 ^{a}	8.14 ± 0.06 ^{a}	7.88 ± 0.03 ^{a}
Lys	8.27 ± 0.63 ^{c}	9.51 ± 0.23 ^{b}	9.77 ± 0.15 ^{b}	10.23 ± 0.01 ^{a}	8.06 ± 0.34 ^{c}	6.39 ± 0.54 ^{d}	7.33 ± 0.29 ^{c}	8.09 ± 0.96 ^{c}
Phe	3.02 ± 0.00 ^{b}	2.96 ± 0.11 ^{b}	2.94 ± 0.01 ^{b}	3.00 ± 0.01 ^{b}	3.44 ± 0.66 ^{ab}	3.96 ± 0.06 ^{a}	3.99 ± 0.08 ^{a}	4.02 ± 0.06 ^{a}
Ser	6.00 ± 0.04 ^{a}	5.85 ± 0.14 ^{ab}	5.75 ± 0.11 ^{b}	5.67 ± 0.01 ^{b}	5.48 ± 0.23 ^{bc}	5.19 ± 0.09 ^{c}	5.10 ± 0.03 ^{c}	5.14 ± 0.07 ^{c}
Thr	5.14 ± 0.00 ^{a}	5.00 ± 0.06 ^{a}	4.98 ± 0.03 ^{a}	5.00 ± 0.01 ^{a}	4.95 ± 0.17 ^{ab}	4.81 ± 0.05 ^{b}	4.73 ± 0.07 ^{b}	4.62 ± 0.03 ^{c}
Tyr	4.02 ± 0.08 ^{c}	4.31 ± 0.33 ^{bc}	4.51 ± 0.00 ^{b}	4.63 ± 0.09 ^{b}	6.01 ± 1.47 ^{a}	7.01 ± 0.15 ^{a}	7.01 ± 0.15 ^{a}	7.98 ± 0.12 ^{a}
Val	6.95 ± 0.05 ^{a}	6.91 ± 0.01 ^{a}	6.72 ± 0.02 ^{a}	6.73 ± 0.08 ^{a}	6.91 ± 0.13 ^{a}	6.87 ± 0.10 ^{a}	6.89 ± 0.01 ^{a}	6.69 ± 0.03 ^{a}
Total EAA	47.12 ± 0.09 ^{d}	48.05 ± 0.11 ^{d}	48.04 ± 0.07 ^{d}	49.04 ± 0.04 ^{c}	50.31 ± 1.09 ^{bc}	51.39 ± 0.12 ^{b}	52.42 ± 0.09 ^{a}	53.13 ±0.17 ^{a}

$^{a-e}$ Different superscripts in the same row denote significant differences ($p < 0.05$); Ala: alanine; Arg: arginine; Asp: aspartic acid; Glu: glutamic acid; Gly: glycine; His: histidine; Ile: isoleucine; Leu: leucine; Lys: lysine; Phe: phenylalanine; Ser: serine; Thr: threonine; Tyr: tyrosine; Val: valine, Total EAA: total essential amino acids.

Table S2. Fatty acid composition of *Tenebrio molitor* larvae of different larval instars (5 to 20) on a dry matter (DM) basis (relative % of total fatty acids) analyzed by GC-FID. Data are presented as mean ± standard deviation, $n = 4$.

Fatty acid	Larvae instars							
(% DM)	5-6	7-8	9-10	11-12	13-14	15-16	17-18	19-20
Myristic acid (C14:0)	2.27 ± 0.13 ^{e}	2.81 ± 0.16 ^{d}	3.16 ± 0.26 ^{c}	3.21 ± 0.15 ^{c}	3.56 ± 0.08 ^{b}	3.41 ± 0.06 ^{bc}	3.37 ± 0.09 ^{c}	4.16 ± 0.05 ^{a}
Palmitic acid (C16:0)	18.45 ± 0.26 ^{a}	18.44 ± 0.44 ^{a}	18.75 ± 0.64 ^{a}	18.91 ± 0.68 ^{a}	19.51 ± 0.31 ^{a}	19.40 ± 0.22 ^{a}	19.06 ± 0.31 ^{a}	18.68 ± 0.81 ^{a}
Palmitoleic acid (C16:1)	1.33 ± 0.00 ^{c}	1.53 ± 0.01 ^{b}	1.29 ± 0.13 ^{c}	1.16 ± 0.12 ^{c}	1.25 ± 0.04 ^{c}	1.25 ± 0.09 ^{c}	1.32 ± 0.04 ^{c}	1.74 ± 0.05 ^{a}

Stearic acid (C18:0)	6.02 ± 0.07 [a]	4.51 ± 0.44 [b]	4.48 ± 0.17 [b]	4.23 ± 0.43 [b]	4.04 ± 0.13 [b]	4.01 ± 0.14 [b]	3.82 ± 0.07 [b]	2.85 ± 0.03 [c]
Oleic acid (C18:1 n-9)	33.57 ± 0.14 [a]	34.01 ± 0.61 [a]	33.46 ± 1.09 [a]	31.66 ± 0.56 [a]	32.57 ± 0.07 [a]	33.21 ± 0.17 [a]	33.97 ± 0.42 [a]	34.44 ± 0.45 [a]
Linoleic acid (C18:2 n-6)	37.84 ± 0.05 [a]	37.88 ± 1.08 [a]	37.89 ± 1.56 [a]	39.68 ± 0.22 [a]	37.98 ± 0.26 [a]	37.64 ± 0.39 [a]	37.31 ± 0.38 [a]	36.91 ± 0.40 [a]
α-Linolenic acid (C18:3 n-3)	0.53 ± 0.03 [c]	0.83 ± 0.20 [bc]	0.97 ± 0.02 [b]	1.14 ± 0.11 [ab]	1.09 ± 0.03 [ab]	1.09 ± 0.04 [ab]	1.14 ± 0.05 [ab]	1.23 ± 0.03 [a]
∑ SFA	26.73 ± 0.45	25.76 ± 1.04	26.39 ± 1.08	26.36 ± 1.26	27.11 ± 0.51	26.82 ± 0.42	26.25 ± 0.47	25.68 ± 0.89
∑ MUFA	34.90 ± 0.15	35.54 ± 0.62	34.75 ± 1.23	32.82 ± 0.68	33.82 ± 0.11	34.46 ± 0.26	35.29 ± 0.47	36.18 ± 0.50
∑ PUFA	38.37 ± 0.08	38.71 ± 1.28	38.86 ± 1.58	40.82 ± 0.33	39.07 ± 0.29	38.72 ± 0.43	38.46 ± 0.44	38.14 ± 0.42

[a-e] Different superscripts in the same row denote significant differences ($p < 0.05$), SFA: saturated fatty acids, MUFA: monounsaturated fatty acids, PUFA: polyunsaturated fatty acids.

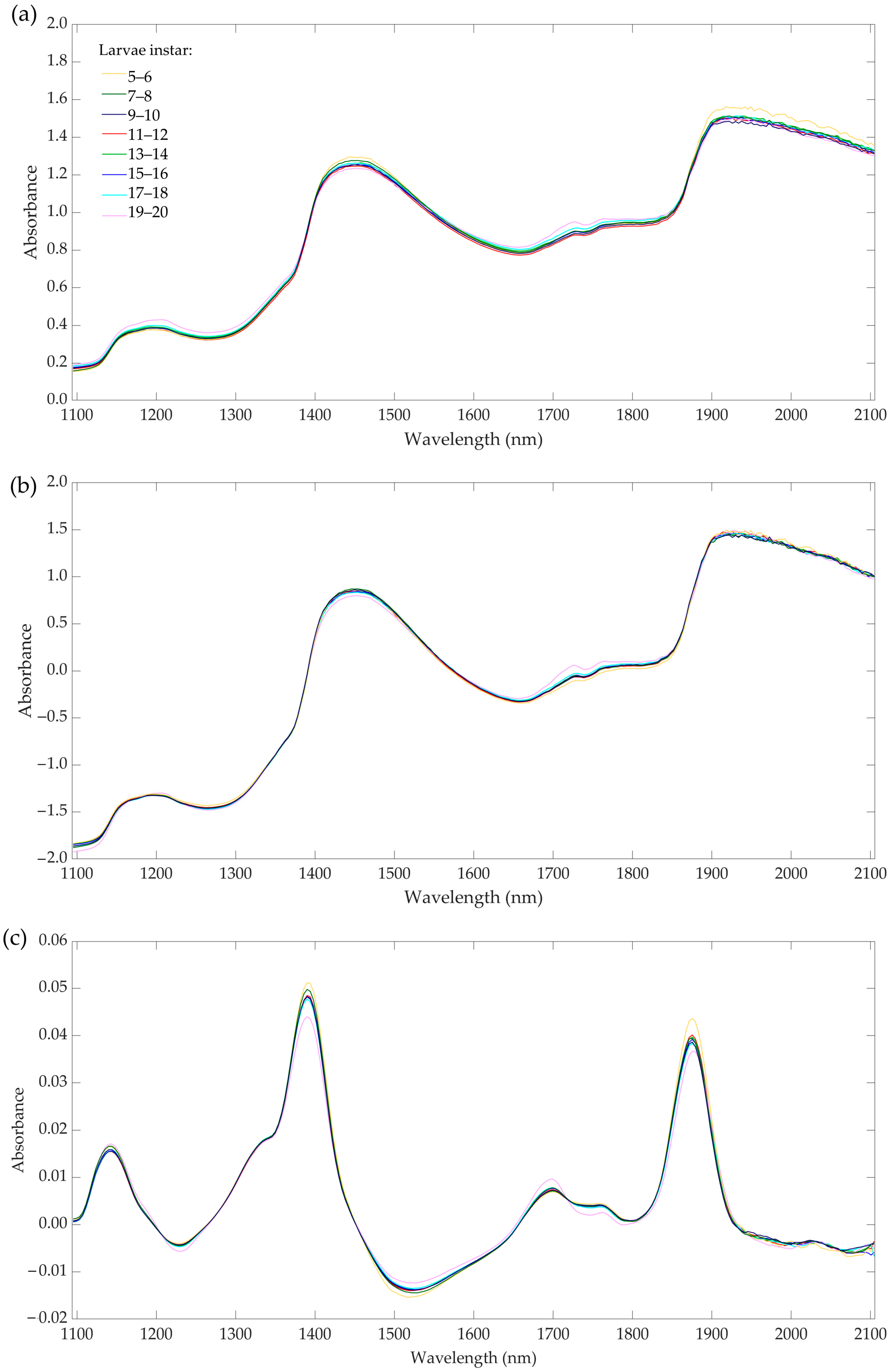

Figure S1. Mean average NIR raw spectra (a) and preprocessed spectra with standard normal variate (b) and first derivative (c) of living *Tenebrio molitor* larvae with different instars 5 to 20 ($n = 10$).
